# Towards Building Blocks for Supramolecular Architectures Based on Azacryptates

**DOI:** 10.3390/molecules25071733

**Published:** 2020-04-09

**Authors:** Ana Miljkovic, Sonia La Cognata, Greta Bergamaschi, Mauro Freccero, Antonio Poggi, Valeria Amendola

**Affiliations:** 1 Department of Chemistry, University of Pavia, V.le Taramelli 12, 27100 Pavia, Italy; ana.miljkovic@unipv.it (A.M.); mauro.freccero@unipv.it (M.F.); antonio.poggi@unipv.it (A.P.); valeria.amendola@unipv.it (V.A.); 2 Istituto di Scienze e Tecnologie Chimiche, National Research Council of Italy, Via M. Bianco 9, 20131 Milano, Italy; greta.bergamaschi@cnr.it

**Keywords:** molecular cages, anion coordination, non-covalent interactions, self-assembled systems, supramolecular architectures

## Abstract

In this work, we report the synthesis of a new bis(tris(2-aminoethyl)amine) azacryptand L with triphenyl spacers. The binding properties of its dicopper complex for aromatic dicarboxylate anions (as TBA salts) were investigated, with the aim to obtain potential building blocks for supramolecular structures like rotaxanes and pseudo-rotaxanes. As expected, UV-Vis and emission studies of [Cu_2_L]^4+^ in water/acetonitrile mixture (pH = 7) showed a high affinity for biphenyl-4,4′-dicarboxylate (dfc^2−^), with a binding constant of 5.46 log units, due to the best match of the anion bite with the Cu(II)-Cu(II) distance in the cage’s cavity. Compared to other similar bistren cages, the difference of the affinity of [Cu_2_L]^4+^ for the tested anions was not so pronounced: conformational changes of L seem to promote a good interaction with both long (e.g., dfc^2−^) and short anions (e.g., terephthalate). The good affinity of [Cu_2_L]^4+^ for these dicarboxylates, together with hydrophobic interactions within the cage’s cavity, may promote the self-assembly of a stable 1:1 complex in water mixture. These results represent a good starting point for the application of these molecular systems as building units for the design of new supramolecular architectures based on non-covalent interactions, which could be of interest in all fields related to supramolecular devices.

## 1. Introduction

Now more than ever, the scientific community, inspired by nature, is exploring new frontiers in the fascinating world of molecular machinery with the aim to induce and control, through specific external stimuli, the mechanical movement of single parts in a molecular/supramolecular assembly [1,2]. Trying to achieve this challenging purpose, beautiful and functional systems controlled for example by light, changes of pH or redox processes were obtained and reported [3,4,5]. Rotaxanes and pseudo-rotaxanes represent an important class of supramolecular architectures: due to the combination of different kinds of interaction between the axle and the ring(s) structure, these molecular devices found a wide range of applications in different fields such as sensing, catalysis, drug delivery, and nanotechnologies [6,7,8,9]. At the basis of their functioning, the meticulous design and engineering of the molecular building blocks are essential because specific interactions are needed to stabilize and control the assembly and the induced movements of the components [10].

In the last twenty years, several authors reported macrocycles as building units in rotaxane and pseudo-rotaxane supramolecular assemblies [11], but the application of molecular cages in this field is not so diffused yet [12]. Some peculiar features of polyamino cage-like systems [13] make them suitable for the application in supramolecular architecture: (1) the easy preparation procedure, based on the Schiff condensation of a polyamine (such as tris(2-aminoethyl)amine, called tren) with a dialdehyde, followed by reduction with NaBH_4_; (2) the high tunability of the shape and the size of the well-defined three-dimensional cavity, through the choice of specific polyamines/dialdehydes in the synthetic step; and (3) the formation of very stable dicopper complexes able to establish strong and specific interactions with bidentate anions. The formation of the so called “cascade” species plays a key role in the recognition process. In fact, the affinity and selectivity for the dianions of interest, like dicarboxylates, principally depends on the good match between the anion bite length and the distance between the free apical positions of the two Cu(II) ions within the cavity. Several papers in the literature regarding bistren cages clearly show that the selectivity of these systems for a specific anion can be regulated by a modulation of the host’s cavity through the opportune change of reagents in the synthetic route [14,15]. This great versatility, associated with an easy design and preparation, represents an important feature for the application of these molecular cages as building units in supramolecular architectures. In a recent paper, we described how the selective interaction between the dicopper complex of a bistren diphenyl azacryptate and the terephthalate anion (previously studied by Fabbrizzi and coworkers [16]) permitted the self-assembly of a pseudo-rotaxane structure in aqueous solution [17]. The presence of two polyoxyethylene chains on the benzene ring of the anion does not interfere with the formation of a stable 1:1 inclusion complex, as demonstrated by the binding constants (logK_11_ values between 5.18 and 4.98). The good fit of the anion bite length within the cavity, together with the intrinsic rigidity of the ligand, ensures a good affinity of terephthalate for the dicopper azacryptate, even if the anion carries long-chain substituents.

**Figure 1:**
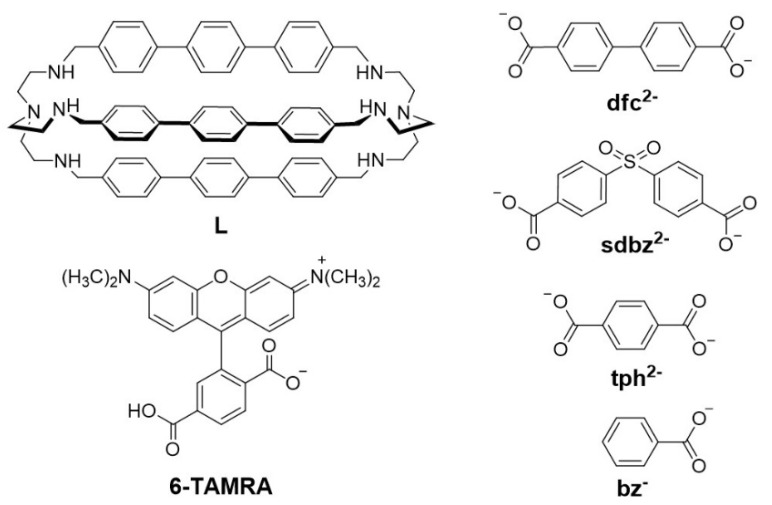
Structures of the azacryptand L, the fluorescent indicator (6-TAMRA) and the carboxylates investigated in this work.

Following this line, we have synthesized a new bigger bistren azacryptand, containing triphenyl spacers, that could interact with longer dicarboxylate anions, with the aim to obtain potential building units for supramolecular architectures. The cage L (reported in Figure 1) was prepared using a straightforward procedure, reported in Supplementary Materials (i.e. SI). As observed for similar bistren-like systems [14,15], the rigid and longer triphenyl spacers are expected to have a positive effect on anion binding selectivity. Moreover, the triphenyl spacers might interact with the hydrophobic groups of the anion, thus favoring the encapsulation in aqueous solutions. For these reasons, the binding properties of the dicopper complex towards anionic substrates were studied in the presence of mono- and dicarboxylates of different shapes and sizes, exhibiting different distances between the carboxylic groups (see Figure 1, biphenyl-4,4′-dicarboxylate, dfc^2−^; 4,4’-sulfonyldibenzoate, sdbz^2−^; terephthalate, tph^2−^; benzoate, bz^−^). The binding properties of [Cu_2_L]^4+^ for all studied anions were investigated through both UV-Vis titrations and competition experiments [18].

## 2. Results and Discussion

Azacryptand L was synthesized from the tren polyamine and [1,1’:4’,1’’-Terphenyl]-4,4’’-dicarboxaldehyde [19] using a known procedure (reported in SI) [20]. L was then complexed with 2 eqv. Cu(II), as the triflate salt, and the compound [Cu_2_L](CF_3_SO_3_)_4_ was isolated and characterized (see SI). The free ligand L was found to be poorly soluble at neutral pH in both aqueous solution and organic solvent:water mixtures, due to the presence of the bulky organic spacers [16]. Consequently, potentiometric studies could not be performed, and the distribution diagram of the ligand at varying pH values is not available. Studies in solution were thus carried out directly on the dicopper complex, [Cu_2_L](CF_3_SO_3_)_4_, in 1:4 (*v*:*v*) water:acetonitrile solution. The pH-spectrophotometric titration of a 0.2 mM solution of [Cu_2_L](CF_3_SO_3_)_4_ (see Figure S1 in SI) pointed out that Cu(II) complexation begins around pH 4 with the development of strong d-d bands between 650 and 850 nm, typical of the trigonal-bipyramidal CuN5 chromophore (i.e., Cu(II) complexes with tripodal bistren-type receptors) [21,22,23,24,25]. Maximum absorbance is reached above pH 6, where the ligand is fully complexed as [Cu_2_L]^4+^. The coordination of a solvent molecule at the apical position of each Cu(II) centre is easily inferred, as it has been observed in the dicopper complexes of similar bistren-like ligands [16,26]. Unfortunately, crystals suitable for X-ray diffraction studies were not available for our system; therefore, this hypothesis could not be verified.

### 2.1. UV-Vis Investigations with Aromatic Dicarboxylates of Different Size/Length

The affinity of [Cu_2_L](CF_3_SO_3_)_4_ towards a series of dicarboxylate anions (as the TBA salts, see Figure 1) was investigated through UV-Vis titrations in 1:4 (*v*:*v*) water:acetonitrile buffered solution [0.02 M 4-(2-hydroxyethyl)-1-piperazineethanesulfonic acid (HEPES buffer), pH 7, T = 25 °C]. In a typical experiment, incremental amounts of a solution of the chosen anion were added to a 50 μM solution of the [Cu_2_L](CF_3_SO_3_)_4_ complex (25 mL, path length = 10 cm), and the corresponding UV-Vis spectra were recorded.

**Figure 2:**
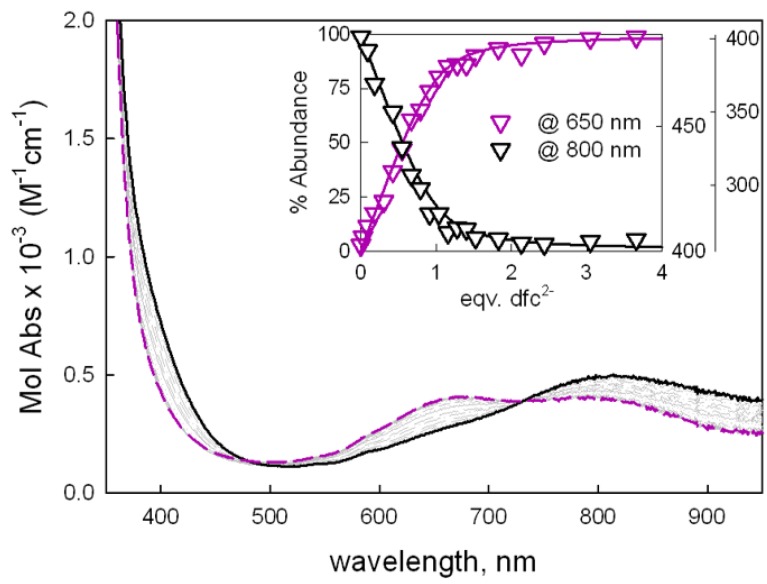
UV-Vis spectra taken upon titration of [Cu_2_L](CF_3_SO_3_)_4_ (50 μM) with dfc^2−^ (as the TBA salt) in H_2_O:CH_3_CN 1:4 at pH 7 (0.02M HEPES buffer; path length = 10 cm). The inset shows the titration profiles at 650 and 800 nm, with the superimposed distribution diagrams of the dicopper complex (C) containing species: purple line, [Cu_2_L(dfc)]^2+^; black line, [Cu_2_L]^4+^; LogK_11_ = 5.46(2).

As already observed for similar metal bistren-cage receptors, higher association constants toward anions could be observed when the guest exhibits the correct bite length (i.e., the distance between two consecutive donor atoms) for bridging the two Cu^II^ centers, without inducing any rearrangement of the cage framework [16,26,27]. For these reasons, considering the high affinity of the reported biphenyl bistren cage toward the terephthalate anion [16], we tested the binding properties of the dicopper(II) complex [Cu_2_L]^4+^ toward longer aromatic dicarboxylates, such as biphenyl-4,4′-dicarboxylate (dfc^2−^) and 4,4’-sulfonyldibenzoate (sdbz^2−^), whose bite length was expected to match better with our new cage’s cavity compared to shorter anions [28]. To confirm this hypothesis, the affinity for smaller terephthalate (tph^2−^) and the monodentate anion benzoate (bz^−^) were also studied: The family of UV-Vis spectra relative to the titration with dfc^2−^, as well as the titration profiles with the superimposed distribution diagrams, are reported in Figure 2 (titrations with sdbz^2−^ and thp^2−^ are reported in the SI, Figures S2 and S3).

Notably, the coordination of (di)carboxylates to the copper ions within the cage was accompanied by a typical change in the relative intensity of the d-d bands, already observed in similar dicopper bistren cryptates. In particular, the band at 810 nm decreased in intensity and shifted towards higher energies, while the absorbance between 670 and 700 nm increased. These spectral variations are typically involved by the change in the coordination geometry around the metal centers due to the coordination of the two carboxylate groups in the apical positions of the copper(II) ions within the cavity [16,17,21,29]. The titration data allowed for defining the stoichiometry of the adducts: the formation of a 1:1 complex was observed with all the investigated dicarboxylate species. Titration data were fitted with the Hyperquad package [30] to estimate equilibrium constants, reported in Table 1.

Among the investigated dicarboxylate anions, binding constants (determined by UV-Vis titrations) decrease along the series dfc^2−^ > sdbz^2−^ > tph^2−^. As expected, the highest affinity was found for dfc^2−^, whose bite seems to better match the distance between Cu(II) ions within the cavity, inducing a slightly endothermic rearrangement during the recognition process. On the other hand, a cavity constraint is expected to occur with the inclusion of smaller anions (tph^2−^) inducing a decrease of the binding constants. For the benzoate anion (bz^−^), only small changes were observed in the UV-Vis spectrum, which could be attributed to a weak interaction between bz^−^ and the cage; however, no binding constants could be obtained from data treatment.

Notably, the inclusion process is mainly enthalpy driven and contrasted by the negative entropy contribution (related to the total decrease of particles and to the organization of the receptor’s framework following anion encapsulation). In general, a bidentate anion offers two distinct donor atoms for coordination, thus allowing the formation of more intense coordinative interactions and reinforcing the enthalpic gain of the complexation process. In addition, dfc^2−^ and sdbz^2−^ could establish very efficient hydrophobic and π–π* interactions, probably reinforcing the coordination process. Furthermore, the coordination of a double negative anion induces a release of water molecules larger than in the case of a monodentate guest. The resulting increase of the translational entropy contribution could favor the recognition process [31].

### 2.2. Anion Binding Studies Using the ID Approach

Anion binding studies were also carried out using the indicator displacement (ID) paradigm successful applied in molecular recognition [16,18,27]. We chose 6-TAMRA (i.e., 6-Carboxytetramethylrhodamine) as a fluorescent indicator due to the presence of a 1,4-phenyldicarboxylate unit on the dye’s core, which could bind to the copper ions within the cage’s cavity. Even if the 1,4-phenyldicarboxylate’s bite is not optimal for our receptor, the interaction of 6-TAMRA with the cage in aqueous solution is expected to be favored by hydrophobic effects and by π–π* interactions between the rhodamine core and the triphenyl spacers [32,33]. Moreover, 6-TAMRA is commercially available and it has been successfully employed for the fluorescent detection of dicarboxylates in water, e.g., using the dicopper azacryptate with diphenyl spacers as the receptor [16,34].

**Figure 3:**
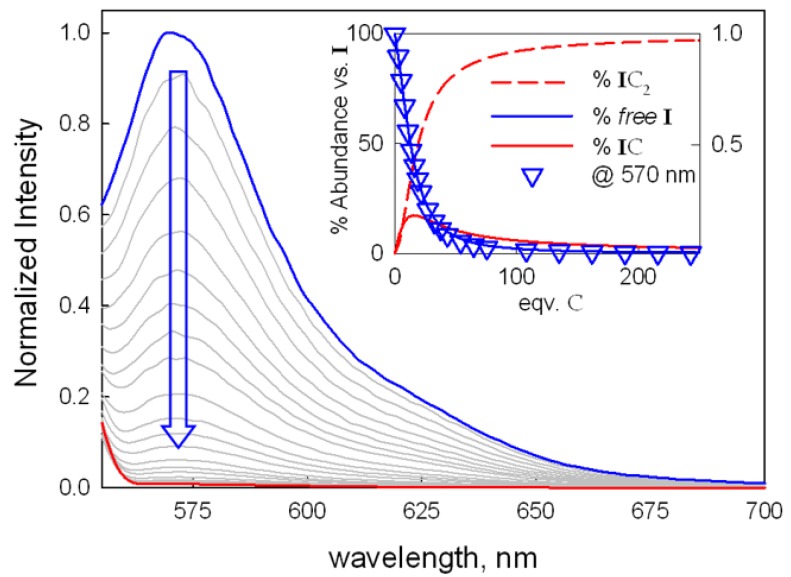
Normalized emission spectra taken upon titration of 6-TAMRA, I (0.25 μM, λ_exc_ = 548 nm) with [Cu_2_L](CF_3_SO_3_)_4_ (C) in H_2_O:CH_3_CN 1:4 at pH 7 (0.02 M HEPES buffer). The inset shows the titration profile as the normalized intensity at 570 nm vs. eqv. of the added complex, with the superimposed distribution diagram of the indicator containing species (I, 6-TAMRA; C, [Cu_2_L]^4+^; IC and IC_2_, 1:1 and 1:2 I:C adducts, respectively).

We first studied the binding of 6-TAMRA to [Cu_2_L](CF_3_SO_3_)_4_ by UV-Vis and fluorimetric titrations in 1:4 water:acetonitrile solution, buffered at pH 7 (0.02 M HEPES buffer). In these conditions, the indicator was fully fluorescent (λ_em_ = 570 nm) and showed an absorption band in the visible region with a maximum at 554 nm. Upon addition of [Cu_2_L](CF_3_SO_3_)_4_, 6-TAMRA underwent a significant quenching process (see Figure 3), while only little changes were observed in the absorption spectrum around 554 nm (see Figure S4). Least-squares treatment of the emission data with the Hyperquad Package [30] allowed the calculation of two equilibrium constants, corresponding to the formation of 1:1 and 1:2 adducts between 6-TAMRA and [Cu_2_L]^4+^ (see IC and IC_2_ in Figure 2; I = 6-TAMRA, C = [Cu_2_L]^4+^). The corresponding equilibria are:

$$I+C⇄{\mathrm{IC},\;\mathrm{LogK}}_{11}=5.06\left(2\right)\;I+2C⇄{\mathrm{IC}}_{2}{,\;\mathrm{LogK}}_{12}=5.82\left(5\right)$$

From these binding constants, we have determined the distribution diagram of the species present at equilibrium over the course of the titration experiment (see the inset in Figure 3). The best model suggested that, at the beginning, the first additions of [Cu_2_L]^4+^ to the solution of the indicator (i.e., in the presence of an excess of I^−^) led to the formation of a 1:1 complex, {[Cu_2_L]—I}^3+^, in which I might be included (or partially included) in the cryptate. With the addition of an excess of C, we observed the formation of the 1:2 adduct {[Cu_2_L]—I—[Cu_2_L]}^7+^, in which 6-TAMRA bridges two Cu(II) centers of two different cryptates. Notably, the formation of adducts with 1:2 I:C stoichiometry had been already observed by our group in the studies of dicopper azacryptate’s binding with both halide anions in pure acetonitrile solution [35] and anionic indicators in methanol:water mixture [27]. As shown in Figure 3, the indicator emission was completely quenched above 100 eqv. of the added complex, i.e., when the indicator is in the IC_2_ form (>90%).

**Figure 4:**
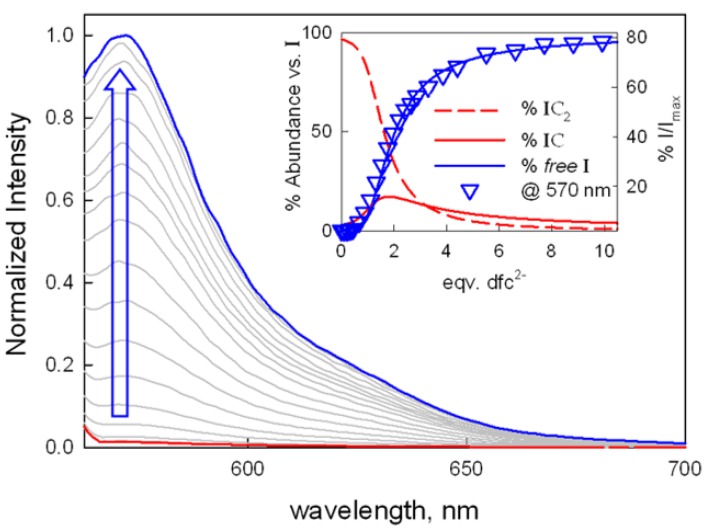
Normalized emission spectra taken upon titration of the chemosensing ensemble solution (0.25 μM I; 50 μM C, λexc = 548 nm) with dfc^2−^ (as the TBA salt) in H_2_O:CH_3_CN 1:4 at pH 7 (0.02 M HEPES buffer). The inset shows the titration profile, as % I/I_max_ (I_max_= emission intensity of I in the absence of C) vs. eqv. of the dicarboxylate anion, with the superimposed distribution diagram of the indicator containing species (I = 6-TAMRA; C = [Cu_2_L]^4+^; IC and IC_2_ = 1:1 and 1:2 I:C adducts, respectively).

For competition assays with dicarboxylate species, a chemosensing ensemble solution was prepared by mixing C (50 μM) and I (0.25 μM) in 1:4 water:acetonitrile at pH 7 (0.02 M HEPES buffer). In these conditions (i.e., in presence of 100 eqv of complex respect to 6-TAMRA), the indicator is bound to the receptor (as the IC_2_ species), and its emission is quenched. Upon titration with dfc^2−^, the indicator is displaced, and the emission intensity is restored up to 80% (see Figure 4). Notably, a full restoration of fluorescence is not obtained with a large excess of the competing dfc^2−^ anion, reasonably due to collisional quenching effects of the dicopper complex on free 6-TAMRA. A similar trend was observed with sdbz^2−^ (see Table 1 and Figure S5). These results confirmed the trend obtained with UV-Vis experiment, with the formation of stable anion:receptor complexes for both dfc^2−^ and sdbz^2−^. In particular, the fitting of the experimental curves for a 1:1 anion:cage equilibrium yielded binding constants with values very close to those determined by UV-Vis titrations (see Table 1).

**Figure 5:**
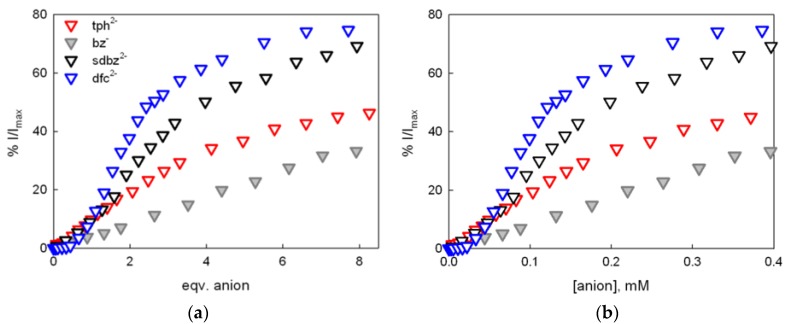
Competition assays with dicarboxylates (as the TBA salts, see the inset symbols) using chemosensing ensemble solutions of C (50 μM) and I (0.25 μM) in 1:4 H_2_O:CH_3_CN at pH 7 (0.02 M HEPES buffer); the families of profiles show the trend of I/I_max_ vs. equivalents of the added anion (**a**) and I/I_max_ vs. anion concentration (**b**).

Competition experiments were also performed with tph^2−^ and bz^−^ (Figures S6 and S7); however, a lower recovery of the indicator emission was obtained (see Figure 5 and Table 1; more details are available in SI). This result is attributable to the lower geometrical complementary between the anion bite and the receptor’s cavity, as well as weaker hydrophobic and π–π* interactions between these anionic guests and the cavity [29]. From the fluorimetric titration profiles, we could determine the binding constants only for dfc^2−^ and sdbz^2−^.

### 2.3. Computational Investigations on the Inclusion Complexes

As already mentioned, single crystals of [Cu_2_L]^4+^ suitable for X-ray diffraction studies could not be obtained. However, some indications on the structures of the inclusion complexes of [Cu_2_L]^4+^ with dicarboxylates were gained through computational investigations.

Preliminary studies were performed on the complex [Cu_2_**L’**(H_2_O)_2_]^4+^ (**L’** = bistren azacryptand with p-xylyl spacers), whose crystal structure is reported in the literature [36]. The calculated structure of [Cu_2_**L’**(H_2_O)_2_]^4+^ in the gas phase, obtained under optimization at the B3LYP/6-31G level of theory (Gaussian09 program package [37]), resulted in being very similar to that in the crystals, as shown by the comparable values of the bond distances (see Figure S8). This result encouraged us to extend the approach to the inclusion complexes of [Cu_2_L]^4+^. In this case, the starting geometry, prepared using GaussView 5.0 was then optimized at the same level of calculation employed for the model complex [Cu_2_**L’**(H_2_O)_2_]^4+^.

**Figure 6:**
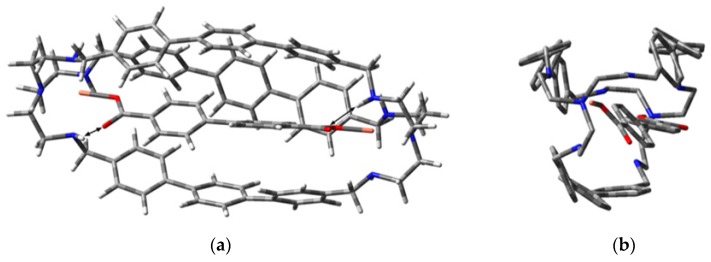
Lateral (**a**) and front (**b**) views of the calculated structure of the complex [Cu_2_L(dfc)]^2+^.

In the most stable conformation of both [Cu_2_L(dfc)]^2+^ (Figure 6) and [Cu_2_L(sdbz)]^2+^ (Figure S9), the Cu(II) ions have a distorted trigonal bipyramidal geometry with Cu···N distances: 2.02 Å (tertiary amine), 2.16, 2.19, 2.38 Å (secondary amines), and the Cu···Cu distance is 14.96 Å. On the other hand, the anion bite (i.e., the separation between the coordinating oxygen atoms on the dianion) is slightly different in the case of dfc^2−^ and sdbz^2−^, being 11.17 Å and 11.14 Å, respectively.

In the preferred conformers of [Cu_2_L(dfc)]^2+^ and [Cu_2_L(sdbz)]^2+^, the anions do not lie in the center of the cavity but slightly outside. However, there is symmetry in coordination because the Cu-carboxylate distance is the same on both sides (1.89 Å for dfc^2−^ and 1.91 Å for sdbz^2−^, respectively). In both [Cu_2_L(dfc)]^2+^ and [Cu_2_L(sdbz)]^2+^, each copper ion is coordinated by a carboxylate unit, bridging together the two Cu(II) ions using only one oxygen for the metal coordination. The distance of the second oxygen atom of the carboxylate group from an amino group of the cage (O···H-N distance 1.88 Å and 1.92 Å for dfc^2−^ and sdbz^2−^, respectively) strongly suggests the existence of a hydrogen bond (indicated with the arrow in Figure 6 and Figure S9).

In the case of terephthalate (see Figure S10), the coordination of the anion to the cage’s cavity is rather asymmetric: the anion is closer to one of the Cu(II) ions (Cu-carboxylate distances: 2.87 Å and 4.99 Å). This result is reasonable considering the small size of this guest with respect to the investigated ones. In particular, the anion bite is only 7.39 Å, therefore too small to bridge the metal centres (dCu···Cu: 14.96 Å).

### 2.4. ESI-MS Characterization of the Inclusion Complexes

Direct experimental evidence of the formation of stable 1:1 complexes between the receptor and the anions (i.e., dfc^2−^ and sdbz^2−^) was provided by the ESI-mass spectrometry studies performed on equimolar solutions of the dicarboxylate anion and [Cu_2_L]^4+^ (30 µM in CH_3_CN:H_2_O 4:1).

**Figure 7:**
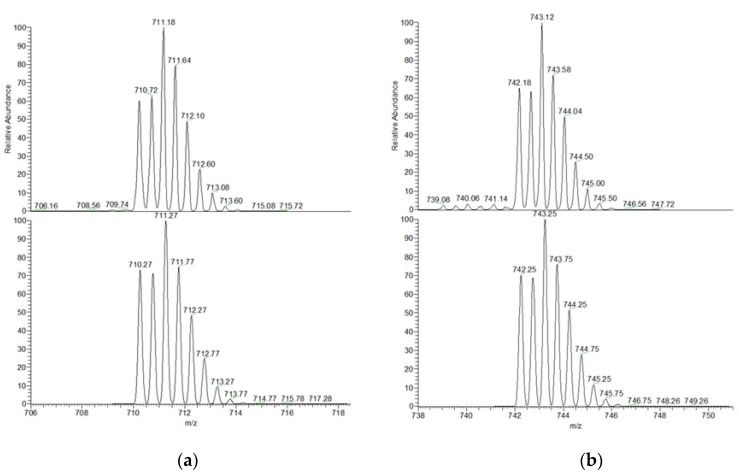
Top: zoom scans of the peaks at 711 m/z and 743 m/z, obtained from the experimental mass spectrum of an equimolar solution of [Cu_2_L]^4+^ and either dfc^2−^ (**a**) or sdbz^2−^ (**b**) in CH_3_CN:H_2_O 4:1. Bottom: simulated peaks, calculated for the double charged adduct [Cu_2_C_86_H_86_N_8_O_4_]^2+^ (**a**) and [Cu_2_C_86_H_86_N_8_O_6_S]^2+^ (**b**).

Figure 7 shows both the experimental and simulated peaks obtained from the ESI-MS analyses of solutions containing the dicopper azacryptate and 1 eqv. of either dfc^2−^ or sdbz^2−^ anions. The comparison with the simulated zoom scan confirmed the attribution of the signals to the inclusion complexes [Cu_2_L(dfc)]^2+^ and [Cu_2_L(sdbz)]^2+^. In the case of other anions, signals attributable to the corresponding adducts could not be found in the in ESI-MS spectra, thus indicating a lower stability of the inclusion species. Experimental and simulated peaks relative to the ESI-MS spectrum of the dicopper complex [Cu_2_L]^4+^ are reported in the SI (Figures S11–S13).

## 3. Materials and Methods

All reagents and solvents were purchased from Sigma-Aldrich and used without further purification. [1,1’:4’,1’’-Terphenyl]-4,4’’-dicarboxaldehyde was synthesized according to a known procedure [19]. In the synthesis of L (reported in SI), NaBH_3_CN was employed as a reducing agent for imine bonds [38]. The solutions used in titrations were prepared from freshly opened solvent bottles. Mass spectra were acquired on a Thermo-Finnigan ion-trap LCQ Advantage Max instrument (Thermo Fischer Scientific, Milano, Italy) equipped with an ESI source, and NMR spectra were recorded on a Bruker ADVANCE 400 spectrometer (operating at 9.37 T, 400 MHz). UV-Vis. spectra were run on a Varian Cary 50 SCAN spectrophotometer (Agilent Technologies Italia, Milano, Italy) with quartz cuvettes of the appropriate path length at 25.0 ± 0.1 °C under inert conditions. Emission spectra were recorded on a PerkinElmer LS 50B instrument (Perkin Elmer Italia, Milano, Italy). Titration data were fitted with the Hyperquad package [30] to determine the equilibrium constants. All the computational studies were carried out using the GAUSSIAN09 program package [37]. The structures were optimized in the gas phase at the B3LYP/6-31 G level of theory.

## 4. Conclusions

In conclusion, a new bistren azacryptand ligand L, characterized by the presence of rigid triphenyl spacers, was synthesized and the binding properties of its dicopper complex with different aromatic dicarboxylates were investigated through spectrophotometric and spectrofluorimetric studies. Least-squares treatment of the experimental data allowed us to determine the binding constants relative to all anions, except for bz^−^ (see Table 1): notably, LogK_11_ values obtained from competition experiments are very close to those obtained from UV-Vis titrations, confirming the affinity trend of our cryptate.

As expected, compared to the biphenyl bistren cage, the larger cavity and the consequent longer Cu(II)—Cu(II) distance in the cryptate L favored the interaction with longer dicarboxylates, such as dfc^2−^ and sdbz^2−^, as demonstrated by LogK_11_ values (reported in Table 1) compared to those relative to tph^2−^ and bz^−^. This binding preference depends on the ability of the anion to bridge the two Cu(II) ions, with coordinative interactions becoming stronger with the matching between the anion bite and the azacryptate’s cavity; in addition, the stronger hydrophobic and π–π* interactions for these anions, compared to tph^2−^ and bz^−^, could give a further contribution to the stability of the 1:1 complex:anion adduct. Moreover, it is important to consider the energetic contribution to the recognition process: in general, the enthalpic gain of the complexation process is higher in the case of more intense coordinative interactions; furthermore, in the presence of a bidentate anion, more water molecules are released during the coordination with the metal centers, with a favorable increase of translation entropy contribution.

However, in contrast with other reported bistren cages [16,29], the difference of the affinity of [Cu_2_L]^4+^ towards the tested anions was not so pronounced. As observed through computational studies, in spite of the rigidity of the spacers, the ligand L seems to adjust its conformational preferences to better interact with long dianions (like dfc^2−^ and sdbz^2−^, that slightly move outside the cavity) and much shorter ones, like thp^2−^ (which coordinates only one Cu(II) center). In any case, the good affinity of [Cu_2_L]^4+^ for the studied anions, in particular for biphenyldicarboxylate, was confirmed through different experimental investigations. The formation of stable 1:1 cage:anion complexes could promote the spontaneous self-assembly of these supramolecular adducts in aqueous mixture, driven not only by positive hydrophobic interactions between the aromatic anions and the cage’s cavity, but also by favorable energetic factors associated with the recognition process. This highlights the significance of size and bond complementarity between receptor and anion and encourages synthetic chemists to design systems of enhanced recognition properties. In accordance with these peculiar features, the results reported in this work could open new possibilities for the application of these molecular systems as building units for the design of new supramolecular architectures based on non-covalent interactions in aqueous solution, which could be of interest in all fields related to supramolecular devices.

## Figures and Tables

**Table 1:** Binding constants obtained by UV-Vis and fluorimetric (indicator displacement, ID) titrations. % I/I_max_ = % recovery of the emission intensity at 570 nm (taken at 8 eqv. of the added anion). Details are reported in the text and in SI. n.a.: not available.

Anion	LogK_11_^UV-Vis^	λ_fin_ UV-Vis (nm)	LogK_11_^ID^	% I/I_max_
dfc^2−^	5.46(3)	670, ~780	5.46(4)	75%
sdbz^2−^	5.08(1)	686, ~780	5.01(1)	70%
tph^2−^	4.43(4)	690(sh.), 800	n.a.	46%
bz^−^	n.a.	n.a.	n.a.	36%

## Supplementary Materials

The following are available online on the journal’s website: Synthesis and characterization of L and [Cu_2_L](CF_3_SO_3_)_4_, Studies in solution, Computational studies, Characterization of [Cu_2_L]^4+^ by ESI-MS, NMR characterization of L.

## References

[1] Bissell R.A., Córdova E., Kaifer A.E., Stoddart J.F. (1994). A chemically and electrochemically switchable molecular shuttle. Nature.

[2] Balzani V., Gómez-López M., Stoddart J.F. (1998). Molecular Machines. Acc. Chem. Res..

[3] Silvi S., Venturi M., Credi A. (2011). Light operated molecular machines. Chem. Commun..

[4] Liu Y., Flood A.H., Bonvallet P.A., Vignon S.A., Northrop B.H., Tseng H.-R., Jeppesen J.O., Huang T.J., Brough B., Baller M. (2005). Linear Artificial Molecular Muscles. J. Am. Chem. Soc..

[5] Erbas-Cakmak S., Leigh D.A., McTernan C.T., Nussbaumer A.L. (2015). Artificial Molecular Machines. Chem. Rev..

[6] Balzani V., Credi A., Venturi M. (2008). Molecular Devices and Machines: Concepts and Perspectives for the Nano World.

[7] Li D., Paxton W.F., Baughman R.H., Huang T.J., Stoddart J.F., Weiss P.S. (2009). Molecular, Supramolecular, and Macromolecular Motors and Artificial Muscles. MRS Bull..

[8] Kay E.R., Leigh D.A. (2015). Rise of the Molecular Machines. Angew. Chem. Int. Ed..

[9] Lewis J.E.M., Galli M., Goldup S.M. (2017). Properties and emerging applications of mechanically interlocked ligands. Chem. Commun..

[10] Kay E.R., Leigh D.A., Zerbetto F. (2007). Synthetic Molecular Motors and Mechanical Machines. Angew. Chem. Int. Ed..

[11] Xue M., Yang Y., Chi X., Yan X., Huang F. (2015). Development of Pseudorotaxanes and Rotaxanes: From Synthesis to Stimuli-Responsive Motions to Applications. Chem. Rev..

[12] Clever G.H., Shionoya M. (2010). A pH Switchable Pseudorotaxane Based on a Metal Cage and a Bis-anionic Thread. Chem. Eur. J..

[13] Lehn J.-M. (1995). Supramolecular Chemistry: Concepts and Perspectives.

[14] Mateus P., Bernier N., Delgado R. (2010). Recognition of anions by polyammonium macrocyclic and cryptand receptors: Influence of the dimensionality on the binding behavior. Coord. Chem. Rev..

[15] Fabbrizzi L. (2018). Cryptands and Cryptates.

[16] Boiocchi M., Bonizzoni M., Fabbrizzi L., Piovani G., Taglietti A. (2004). A Dimetallic Cage with a Long Ellipsoidal Cavity for the Fluorescent Detection of Dicarboxylate Anions in Water. Angew. Chem. Int. Ed..

[17] Amendola V., Miljkovic A., Legnani L., Toma L., Dondi D., Lazzaroni S. (2018). Self-Assembly of Pseudorotaxane Structures from a Dicopper(II) Molecular Cage and Dicarboxylate Axles. Inorg. Chem..

[18] Wiskur S.L., Ait-Haddou H., Lavigne J.J., Anslyn E.V. (2001). Teaching Old Indicators New Tricks. Acc. Chem. Res..

[19] Bounos G., Ghosh S., Lee A.K., Plunkett K.N., DuBay K.H., Bolinger J.C., Zhang R., Friesner R.A., Nuckolls C., Reichman D.R. (2011). Controlling Chain Conformation in Conjugated Polymers Using Defect Inclusion Strategies. J. Am. Chem. Soc..

[20] Jazwinski J., Lehn J.M., Lilienbaum D., Ziessel R., Guilhem J., Pascard C. (1987). Polyaza Macrobicyclic Cryptands: Synthesis, Crystal Structures of a Cyclophane Type Macrobicyclic Cryptand and of Its Dinuclear Copper(I) Cryptate, and Anion Binding Features. J. Chem. Soc. Chem. Commun..

[21] Duggan M., Ray N., Hathaway B., Tomlinson G., Brint P., Pelin K. (1980). Crystal structure and electronic properties of ammine[tris(2-aminoethyl)amine]copper(II) diperchlorate and potassium penta-amminecopper(II) tris(hexafluorophosphate). J. Chem. Soc. Dalton Trans..

[22] Mateus P., Delgado R., Brandão P., Félix V. (2011). Recognition of Oxalate by a Copper(II) Polyaza Macrobicyclic Complex. Chem. A Eur. J..

[23] Thaler F., Hubbard C.D., Heinemann F.W., van Eldik R., Schindler S., Fábián I., Dittler-Klingemann A.M., Hahn F.E., Orvig C. (1998). Structural, Spectroscopic, Thermodynamic and Kinetic Properties of Copper(II) Complexes with Tripodal Tetraamines. Inorg. Chem..

[24] Hathaway B.J., Billing D.E. (1970). The electronic properties and stereochemistry of mono-nuclear complexes of the copper(II) ion. Coord. Chem. Rev..

[25] Dudley R.J., Hathaway B.J., Hodgson P.G., Power P.C., Loose D.J. (1974). Single-crystal electronic and electron spin resonance spectra of four trigonal bipyramidal copper( II ) complexes. J. Chem. Soc. Dalton Trans..

[26] Alibrandi G., Amendola V., Bergamaschi G., Fabbrizzi L., Licchelli M. (2015). Bistren cryptands and cryptates: Versatile receptors for anion inclusion and recognition in water. Org. Biomol. Chem..

[27] Amendola V., Bergamaschi G., Buttafava A., Fabbrizzi L., Monzani E. (2010). Recognition and Sensing of Nucleoside Monophosphates by a Dicopper(II) Cryptate. J. Am. Chem. Soc..

[28] Amendola V., Bergamaschi G., Fabbrizzi L., Licchelli M., Mangano C. (2016). The interaction of Mozobil ^TM^ with carboxylates. Org. Biomol. Chem..

[29] Mateus P., Delgado R., André V., Duarte M.T. (2015). Dicarboxylate Recognition Properties of a Dinuclear Copper(II) Cryptate. Inorg. Chem..

[30] Gans P., Sabatini A., Vacca A. (1996). Investigation of equilibria in solution. Determination of equilibrium constants with the HYPERQUAD suite of programs. Talanta.

[31] Boiocchi M., Bonizzoni M., Ciarrocchi C., Fabbrizzi L., Invernici M., Licchelli M. (2018). Anion Recognition in Water, Including Sulfate, by a Bicyclam Bimetallic Receptor: A Process Governed by the Enthalpy/Entropy Compensatory Relationship. Chem. A Eur. J..

[32] Marcotte N., Taglietti A. (2003). Transition-metal-based Chemosensing Ensembles: ATP Sensing in Physiological Conditions. Supramol. Chem..

[33] McCleskey S.C., Metzger A., Simmons C.S., Anslyn E.V. (2002). Competitive indicator methods for the analysis of citrate using colorimetric assays. Tetrahedron.

[34] Merli D., La Cognata S., Balduzzi F., Miljkovic A., Toma L., Amendola V. (2019). A smart supramolecular device for the detection of t,t-muconic acid in urine. New J. Chem..

[35] Amendola V., Bergamaschi G., Boiocchi M., Fabbrizzi L., Poggi A., Zema M. (2008). Halide ion inclusion into a dicopper(II) bistren cryptate containing ‘active’ 2,5-dimethylfuran spacers: The origin of the bright yellow colour. Inorg. Chim. Acta.

[36] Yang L.-Z., Li Y., Zhuang X.-M., Jiang L., Chen J.-M., Luck R.L., Lu T.-B. (2009). Mechanistic Studies of C-C Bond Cleavage of Nitriles by Dinuclear Metal Cryptates. Chem. Eur. J..

[37] Frisch M.J., Trucks J.W., Schlegel H.B., Scuseria G.E., Robb M.A., Cheeseman J.R., Scalmani G., Barone V., Mennucci B., Petersson G.A. (2009). Gaussian 09.

[38] Borch R.F., Bernstein M.D., Durst H.D. (1971). Cyanohydridoborate anion as a selective reducing agent. J. Am. Chem. Soc..

