# Supplementary material for: Towards Building Blocks for Supramolecular Architectures Based on Azacryptates
Source: Molecules. 2020 Apr 9;25(7):1733. doi: 10.3390/molecules25071733 (PMC7181268; doi:10.3390/molecules25071733)
Supplement: Supplementary file 1 [file molecules-25-01733-s001.pdf]

molecules

SUPPLEMENTARY INFORMATION

Towards Building Blocks for Supramolecular Architectures Based on Azacryptates

Ana Miljkovic[1], Sonia La Cognata[1,*], Greta Bergamaschi[2], Mauro Freccero[1], Antonio Poggi[1] and Valeria Amendola[1]

[1]Department of Chemistry, Università degli Studi di Pavia, via Taramelli 12, 27100, Pavia (Italy);
[2] Consiglio Nazionale Delle Ricerche, Istituto di Chimica Del Riconoscimento Molecolare (ICRM), via M. Bianco 9, 20131, Milano (Italy).
* Correspondence: sonia.lacognata01@universitadipavia.it

Index:

Contents

Index: 1
1. Synthesis 2
 1.1. Synthesis of L 2
 1.2. Synthesis of $[Cu_2L](CF_3SO_3)_4$ 2
2. Studies in Solution 3
 2.1. pH-Spectrophotometric Titration of $[Cu_2L](CF_3SO_3)_4$ 3
 2.2. UV-Vis Titrations of $[Cu_2L](CF_3SO_3)_4$ 3
 2.3 Emission Studies 5
3. Computational Studies 7
4. Characterization of $[Cu_2L]^{4+}$ by ESI-MS 9
5. NMR Characterization of L 12
References 15

1. Synthesis

All reagents and solvents were purchased from Sigma-Aldrich and used without further purification. [1,1':4',1''-Terphenyl]-4,4''-dicarboxaldehyde was synthesized according to a known procedure [1]. In the synthesis of L, $NaBH_3CN$ was employed a reducing agent for imine bonds [2].

1.1. Synthesis of L

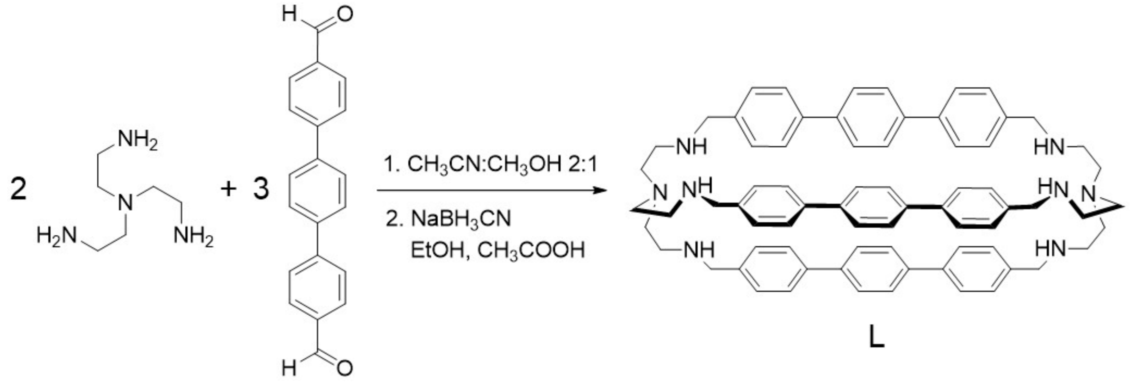

One-hundred milligrams of [1,1':4',1''-Terphenyl]-4,4''-dicarboxaldehyde (100 mg, 0.35 mmol) in 60 mL of 2:1 $CHCl_3$:MeOH were added to a solution of tren (36 ul, 0.35 mmol) in 120 mL of the same medium. The reaction mixture was stirred for 24 hrs at RT, before taking to dryness. The solid residue was suspended in 100 mL EtOH and dissolved by addition of CH_3COOH. The imine bonds are reduced by addition of $NaBH_3CN$ (3 eqv. per C=N) to the mixture at RT. The solution is then stirred at 50 °C overnight. After basification with excess NaOH(aq), EtOH is evaporated and the aqueous layer is extracted with $CHCl_3$ (10 × 25 mL). The reunited organic phases are dried over anhydrous Na_2SO_4. After evaporation, a white solid is obtained. Yield: 70%. ^{1}H-NMR (d_6-DMSO + 1M CF_3SO_3H in D_2O, 400 MHz) δ 7.61 (12H, d, H-**a**), 7.49 (12H, s, H-**c**), 7.34 (12H, d, H-**b**), 4.20 (12H, s, H-**3**), 3.12 (12H, s, H-**2**), 2.76 (12H, s, H-**1**). ^{13}C-NMR (d_6-DMSO + 1M CF_3SO_3H in D_2O, 100 MHz), δ 139.04 (6C, C-**q2**), 137.44 (6C, C-**q3**), 130.04 (6C, C-**q1**), 129.69 (12C, C-**b**), 126.37-126-14 (24C, C-**a**, C-**c**), 48.99 (12C, C-**3**), 48.62 (12C, C-**1**), 42.70 (12C, C-**2**). ESI-MS (MeOH) *m/z* 528.7 $[M+2H]^{2+}$. Elemental analysis calculated for $C_{72}H_{78}N_8$ C, 81.93; H, 7.45; N, 10.62%. Found: C, 81.85; H, 7.63; N, 10.52%.

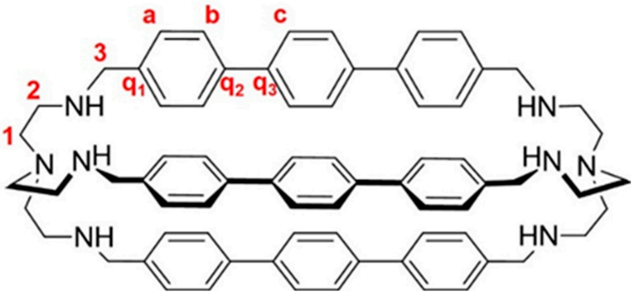

1.2. Synthesis of $[Cu_2L](CF_3SO_3)_4$

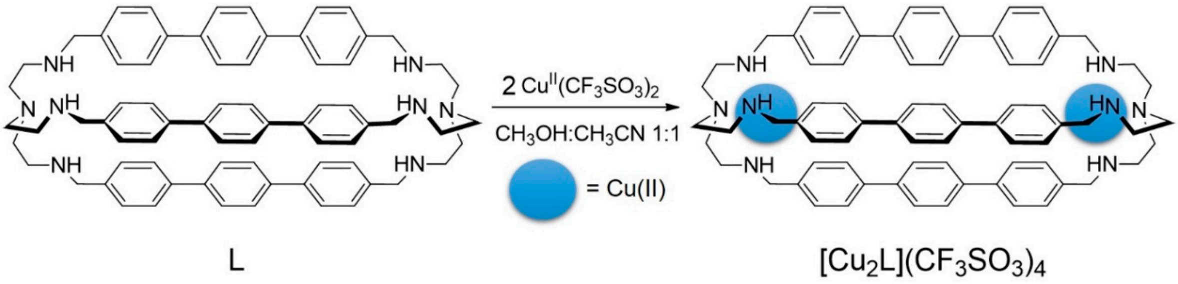

To begin, 55.1 mg (0.052 mmol) of L are suspended in 40 mL of MeOH:CH_3CN 1:1 (*v/v*) mixture. Then, 38.3 mg $Cu^{II}(CF_3SO_3)_2$ (0.107 mmol) in 5 mL MeOH are added, and the obtained solution is refluxed for 3 hrs. After filtering, the solvent is evaporated, and the solid residue is treated with Et_2O. A light green powder is obtained. Yield: 67%. ESI-MS (MeOH) *m/z* 590.22 $[Cu^{II}_2(L\text{-}2H^+)]^{2+}$, *m/z* 665.16 $[Cu^{II}_2(L\text{-}H+CF_3SO_3)]^{2+}$ and *m/z* 740.04 $[Cu^{II}_2L(CF_3SO_3)_2]^{2+}$. Elemental analysis calculated for $[Cu^{II}_2(C_{72}H_{78}N_8)(CF_3SO_3)_4]\cdot 1H_2O$ C, 50.80; H, 4.48; N, 6.24%. Found: C, 50.57; H, 4.59; N, 6.35%.

2. Studies in Solution

2.1. pH-Spectrophotometric Titration of $[Cu_2L](CF_3SO_3)_4$

pH-spectrophotometric titrations were performed on a 0.2 mM solution of $[Cu_2L](CF_3SO_3)_4$ in CH_3CN:H_2O 4:1 mixture ($NaCF_3SO_3$ 0.05 M), 25 °C. In a typical titration, aliquots of carbonate-free standard 0.1 M NaOH were added to the solution of the azacryptate, both the electrochemical potential and the UV-Vis spectrum of the solution were recorded after each addition. Prior to the titration, the standard electrochemical potential (E°) was determined by the Gran method [3].

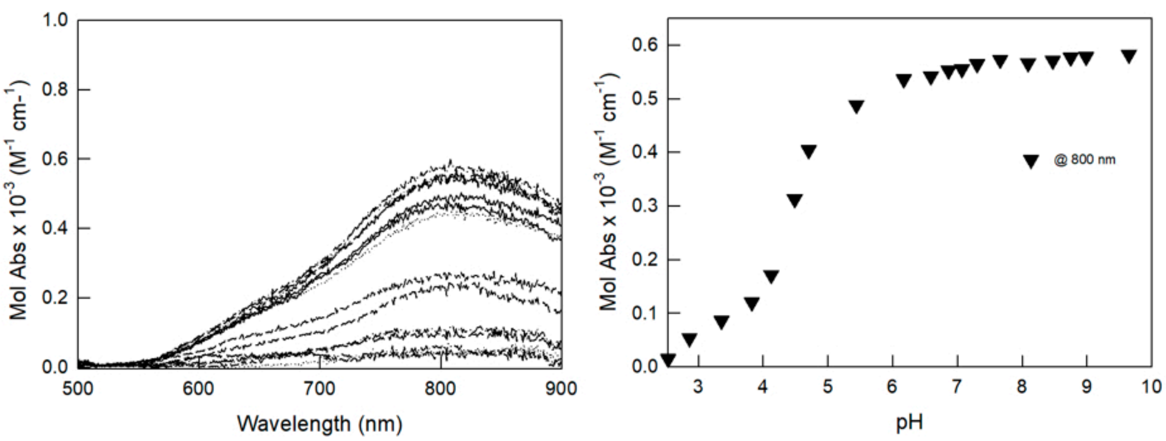

Figure S1. pH-spectrophotometric titration (left) and profile at 800 nm (right) of $[Cu_2L](CF_3SO_3)_4$.

2.2. UV-Vis Titrations of $[Cu_2L](CF_3SO_3)_4$

UV-Vis titrations of $[Cu_2L](CF_3SO_3)_4$ with biphenyl-4,4′-dicarboxylate (dfc^{2-}), 4,4'-sulfonyldibenzoate ($sdbz^{2-}$), terephthalate (tph^{2-}) and benzoate (bz^-) TBA salts were performed in 1:4 (v:v) water:acetonitrile buffered solution (0.02 M HEPES buffer, pH 7, T = 25 °C). In a typical experiment, incremental amounts of a solution of the chosen anion were added to a 50 µM solution of the $[Cu_2L](CF_3SO_3)_4$ complex (10 cm quartz cuvette, 25 ml, path length = 10 cm), and the corresponding UV-Vis spectra were recorded using a Varian Cary 50 SCAN spectrophotometer. Titration data were fitted with the Hyperquad package [4] to estimate equilibrium constants.

The same procedure was followed for the UV-Vis titration of the fluorescent indicator 6-carboxytetramethylrhodamine (6-TAMRA, 0.25 µM) with $[Cu_2L](CF_3SO_3)_4$ in 1:4 (*v/v*) H_2O:CH_3CN buffered solution (0.02 M HEPES buffer) in a 1 cm quartz cuvette.

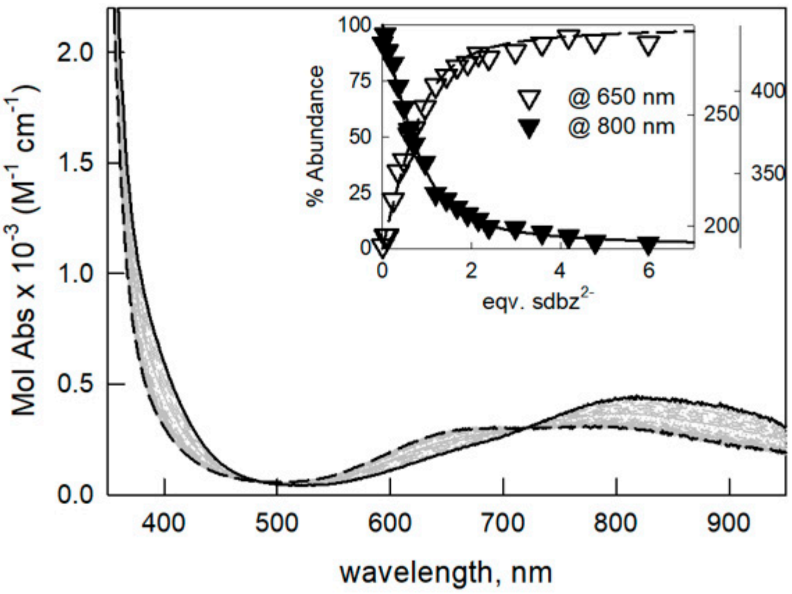

Figure S2. UV-Vis spectra taken upon titration of $[Cu_2L](CF_3SO_3)_4$ (50μM) with $sdbz^{2-}$ (as the TBA salt) in H_2O:CH_3CN 1:4 at pH 7 (0.02 M HEPES buffer; path length = 10 cm). The inset shows the titration profiles at 650 and 800 nm, with the superimposed distribution diagram of the dicopper complex containing species: dotted line, $[Cu_2L(sdbz)]^{2+}$; solid line, $[Cu_2L]^{4+}$; $LogK_{11}$ = 5.08(1).

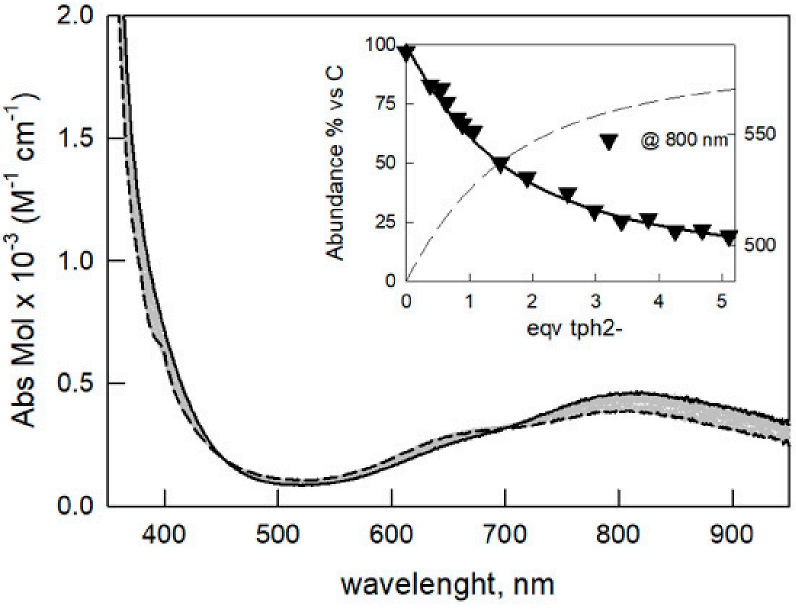

Figure S3. UV-Vis spectra taken upon titration of $[Cu_2L](CF_3SO_3)_4$ (50 μM) with tph^{2-} (as the TBA salt) in H_2O:CH_3CN 1:4 at pH 7 (0.02 M HEPES buffer; path length = 10 cm). The inset shows the titration profiles at 800 nm, with the superimposed distribution diagram of the dicopper complex containing species: dotted line, $[Cu_2L(tph)]^{2+}$; solid line, $[Cu_2L]^{4+}$; $LogK_{11}$ = 4.43(4).

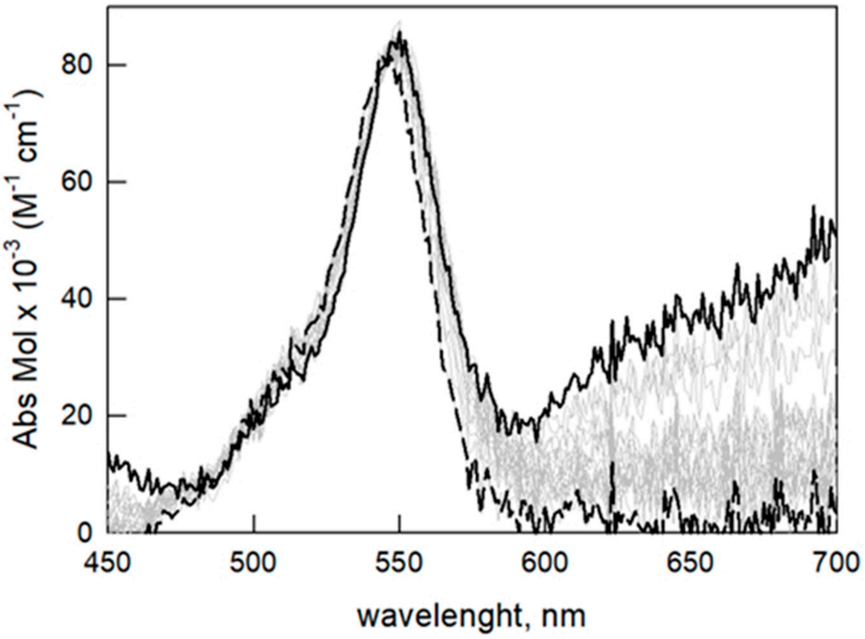

Figure S4. Family of UV-Vis spectra recorded during the titration of 6-TAMRA (0.25 μM, path length = 1 cm) with $[Cu_2L](CF_3SO_3)_4$ in H_2O:CH_3CN 1:4 at pH 7 (0.02 M HEPES buffer).

2.3 Emission Studies

Emission spectra were recorded on a PerkinElmer LS 50B instrument. Fluorimetric titration of 6-TAMRA (I, λ_{exc} = 548 nm) with $[Cu_2L](CF_3SO_3)_4$ (C) were performed in 1:4 H_2O:CH_3CN buffered solution (0.02 M HEPES buffer, pH 7) in a 1 cm quartz cuvette: an incremental amount of the complex solution was added to a 0.25 μM solution of indicator (stored in the dark) and the corresponding emission spectra were recorded. The fluorescent intensity was normalized at 570 nm.

For competition assays with dicarboxylate species, a chemosensing ensemble solution was prepared by mixing C (50 μM) and I (0.25 μM) in 1:4 H_2O:CH_3CN buffered solution (0.02 M HEPES buffer, pH 7). In these conditions, the emission of 6-TAMRA was totally quenched (fluorescence intensity normalized at 570 nm). An increasing amount of a solution of the chosen anion was added to the chemosensing ensemble solution and the corresponding emission spectra were recorded. Titration data were fitted with the Hyperquad package [4] to estimate equilibrium constants.

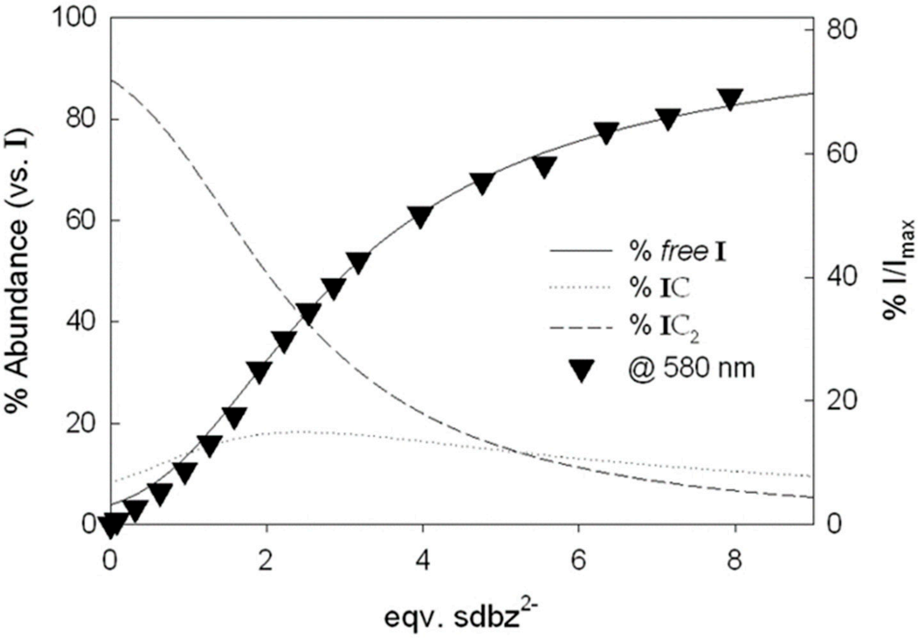

Figure S5. Fluorimetric titration of the chemosensing ensemble solution (0.25 μM I; 50 μM C, λ_{exc} = 548 nm) with sdbz^{2-} (as the TBA salt) in H_2O:CH_3CN 1:4 at pH 7 (0.02 M HEPES buffer). The titration profile (left), as % I/I_{max} (I_{max}= emission intensity of I in the absence of C) vs. eqv. of the dicarboxylate anion, with the superimposed distribution diagram of the indicator containing species (I = 6-TAMRA; C = $[Cu_2L]^{4+}$; IC and IC_2 = 1:1 and 1:2 I:C adducts, respectively). The distribution diagram was obtained using the equilibrium constants reported in Table 1 and in the text.

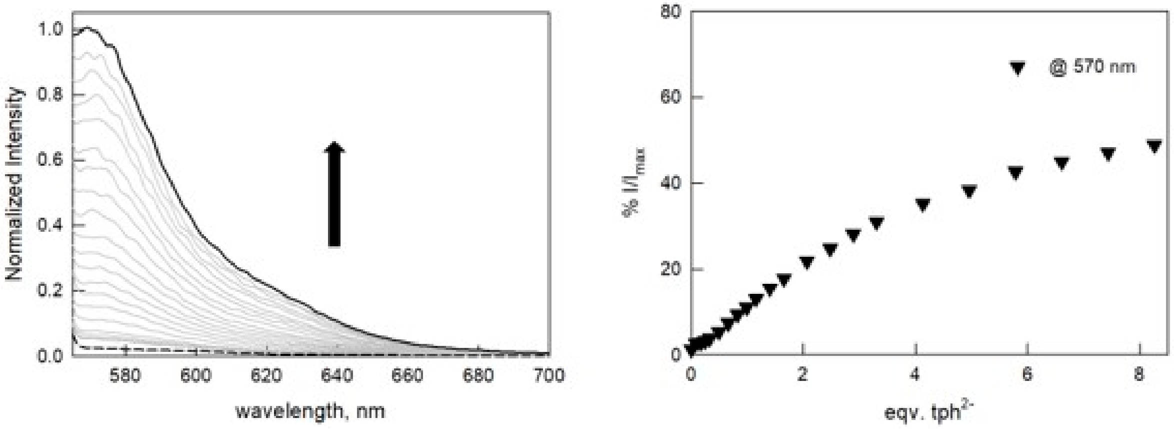

Figure S6. Normalized emission spectra (right) taken upon titration of the chemosensing ensemble solution (0.25 μM **I**; 50 μM C, λ_{exc} = 548 nm) with tph^{2-} (as the TBA salt) in H_2O:CH_3CN 1:4 at pH 7 (0.02 M HEPES buffer). The titration profile (left), as % I/I_{max} (I_{max}= emission intensity of **I** in the absence of C) vs. eqv. of the dicarboxylate anion.

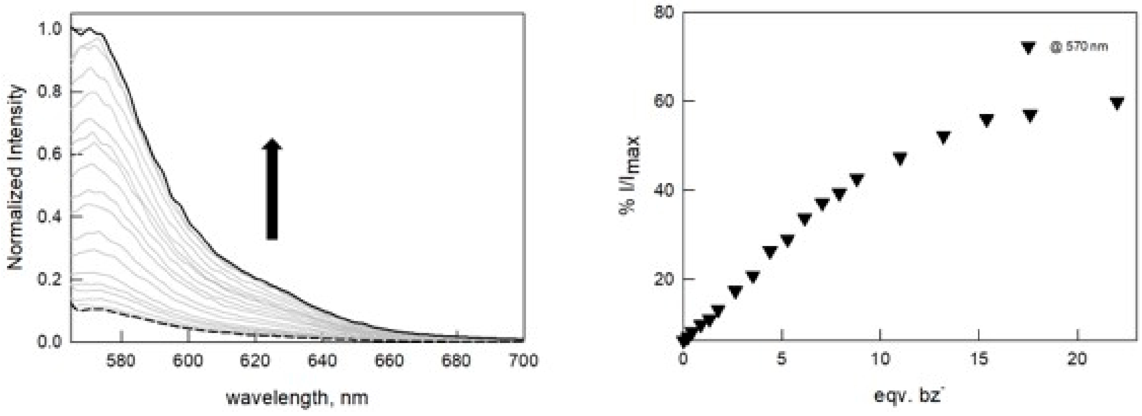

Figure S7. Normalized emission spectra (right) taken upon titration of the chemosensing ensemble solution (0.25 μM **I**; 50 μM C, λ_{exc} = 548 nm) with bz^- (as the TBA salt) in H_2O:CH_3CN 1:4 at pH 7 (0.02 M HEPES buffer). The titration profile (left), as % I/I_{max} (I_{max}= emission intensity of **I** in the absence of C) vs. eqv. of the dicarboxylate anion.

3. Computational Studies

All the computational studies were carried out using the GAUSSIAN09 program package [5]. The structures were optimized in the gas phase at the B3LYP/6-31 G level of theory.

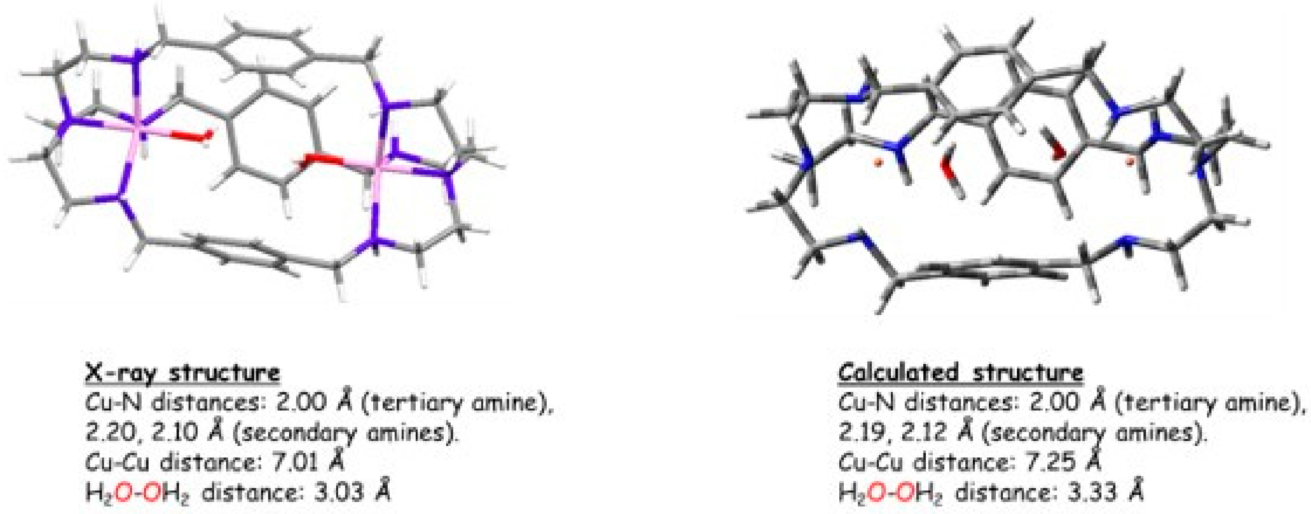

Figure S8. Comparison between crystal and calculated structures for the complex $[Cu_2\mathbf{L'}(H_2O)_2]^{4+}$ (**L'** = bistren azacryptand with p-xylyl spacers).

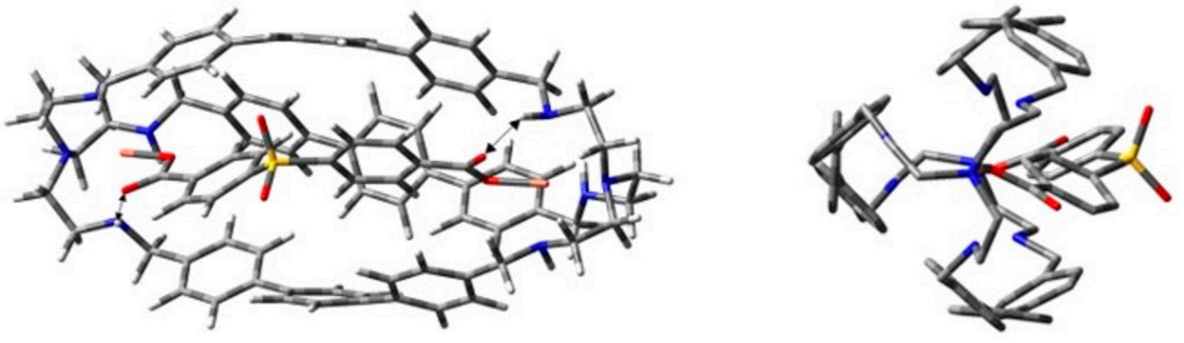

(a) **(b)**

Figure S9. Lateral (**a**) and front (**b**) view of the calculated structure of the complex $[Cu_2L(sdbz)]^{2+}$.

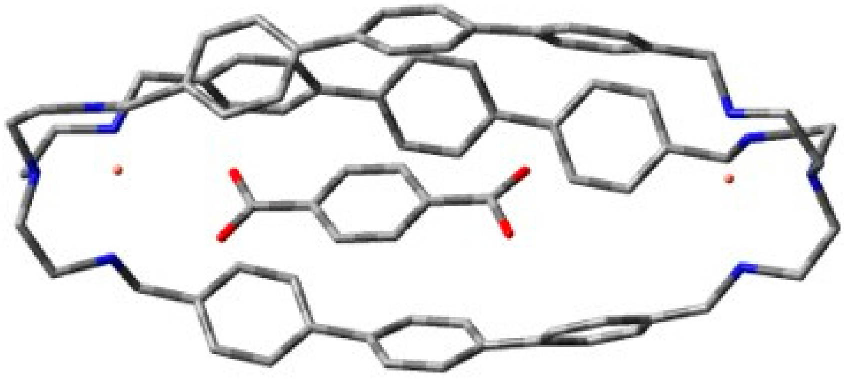

Figure S10. Lateral view of the calculated structure of the complex $[Cu_2L(tph)]^{2+}$.

4. Characterization of $[Cu_2L]^{4+}$ by ESI-MS

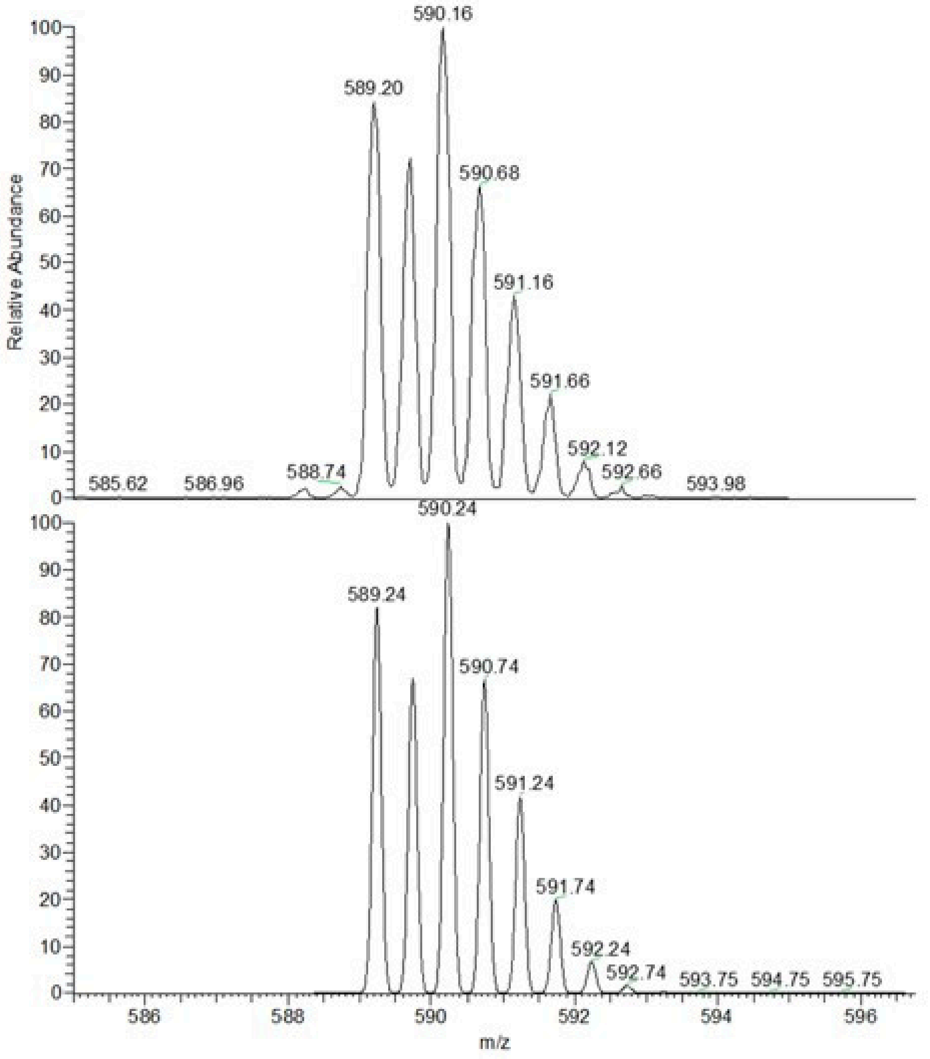

Figure S11. Top: zoom scan of the peak at 590 *m*/*z*, obtained from the experimental ESI-MS spectrum of a solution of $[Cu_2L(CF_3SO_3)_4]$ in CH_3CN:H_2O 4:1. **Bottom:** simulated peak, calculated for the species $[Cu_2C_{72}H_{76}N_8]^{2+}$ (i.e., $[Cu^{II}_2(L\text{-}2H^+)]^{2+}$).

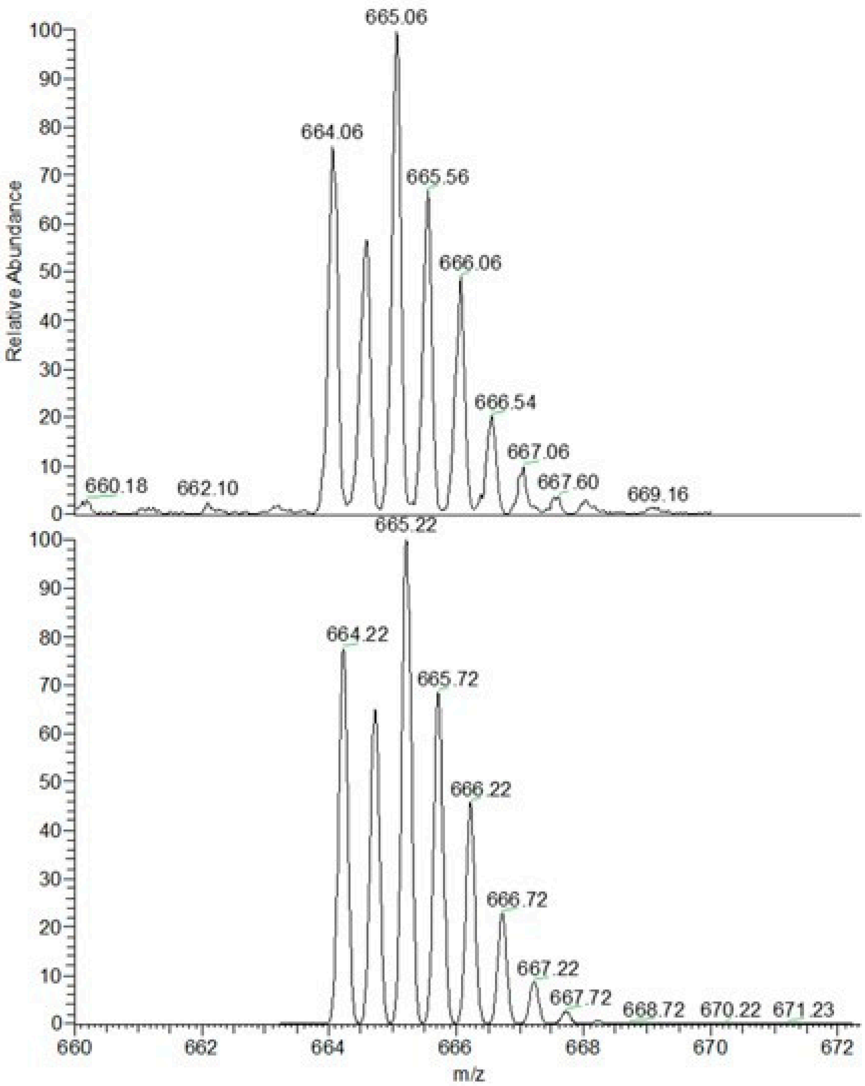

Figure S12. Top: zoom scan of the peak at 665 *m*/*z*, obtained from the experimental ESI-MS spectrum of a solution of [$Cu_2L(CF_3SO_3)_4$] in CH_3CN:H_2O 4:1. **Bottom:** simulated peak, calculated for the species [$Cu_2C_{73}H_{77}N_8O_3F_3S$]$^{2+}$ (i.e., [Cu^{II}_2(L-H+CF_3SO_3)]$^{2+}$).

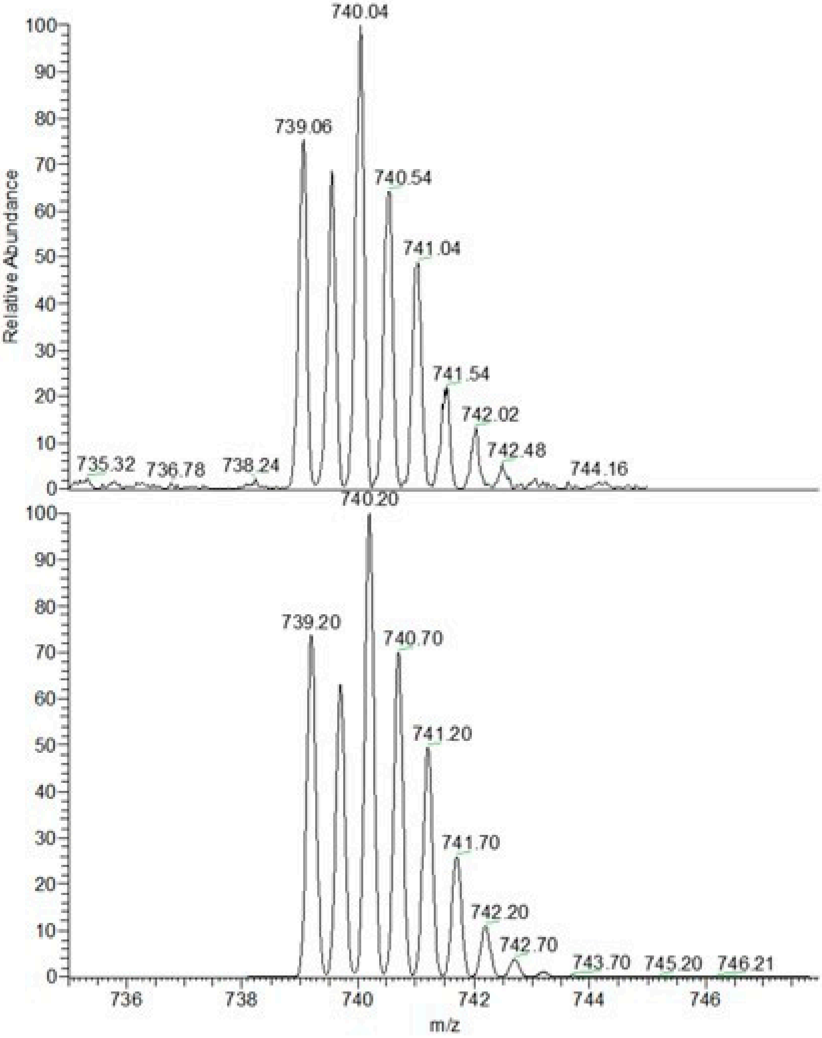

Figure S13. Top: zoom scan of the peak at 740 *m*/*z*, obtained from the experimental ESI-MS spectrum of a solution of $[Cu_2L(CF_3SO_3)_4]$ in $CH_3CN:H_2O$ 4:1. **Bottom:** simulated peak, calculated for the species $[Cu_2C_{74}H_{78}N_8O_6F_6S_2]^{2+}$ (i.e., $[Cu^{II}_2L(CF_3SO_3)_2]^{2+}$).

In the case of dicopper complex, the ESI-MS studies revealed the presence of three double-charged species, in which both copper centers are in the +2 oxidation state, but in the first case (peak at 590 *m*/*z*) the ligand is doubly deprotonated ($[Cu^{II}_2(L\text{-}2H^+)]^{2+}$), while in the second case (665 *m*/*z*) is once deprotonated ($[Cu^{II}_2(L\text{-}H^+CF_3SO_3)]^{2+}$). Notably, this process (i.e., ligand deprotonation) is common in the ESI-MS spectra of copper complexes with polyamine ligands, especially when using MeOH as solvent [6].

5. NMR Characterization of L

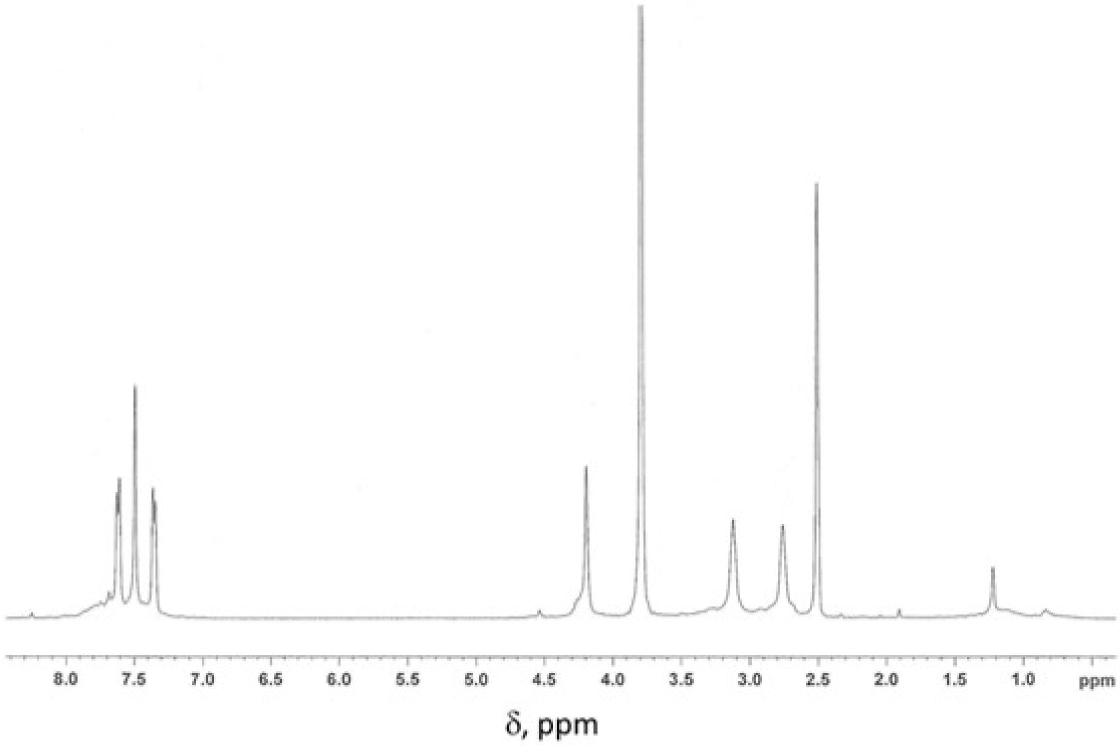

Figure S14. ^{1}H–NMR spectra of L in d_6-DMSO + 1M CF_3SO_3H in D_2O.

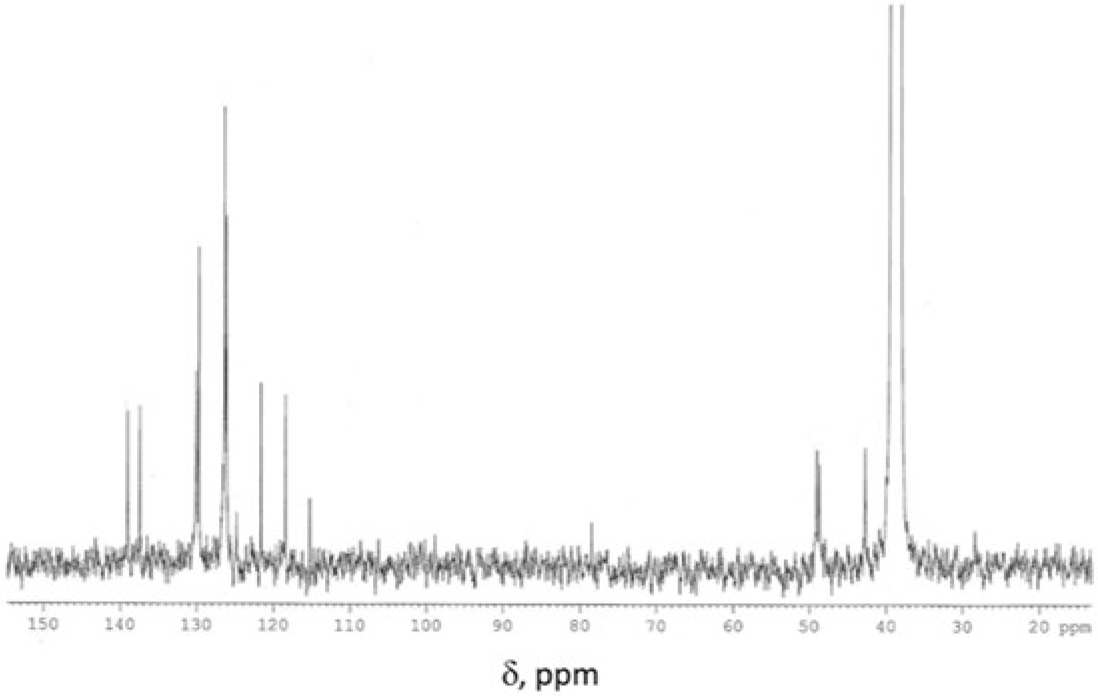

Figure S15. ^{13}C–NMR spectra of L in d_6-DMSO + 1M CF_3SO_3H in D_2O.

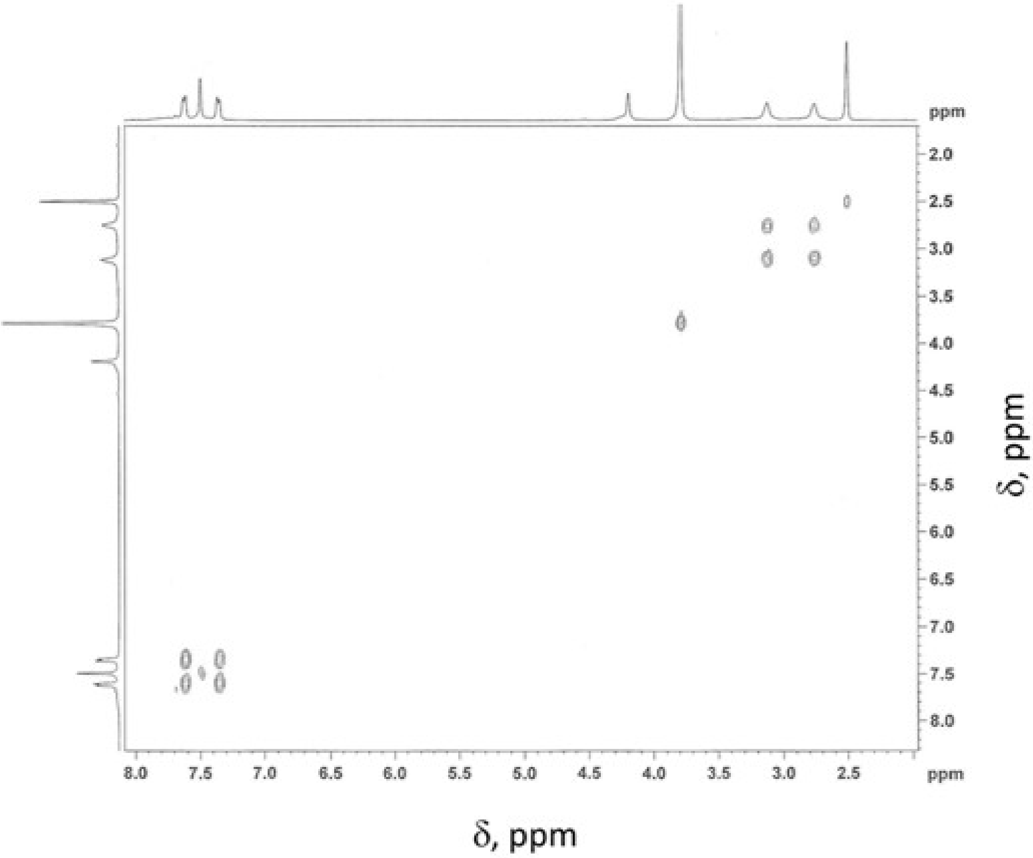

Figure S16. ^{1}H– ^{1}H COSY spectrum of L in d_6-DMSO + 1M CF_3SO_3H in D_2O.

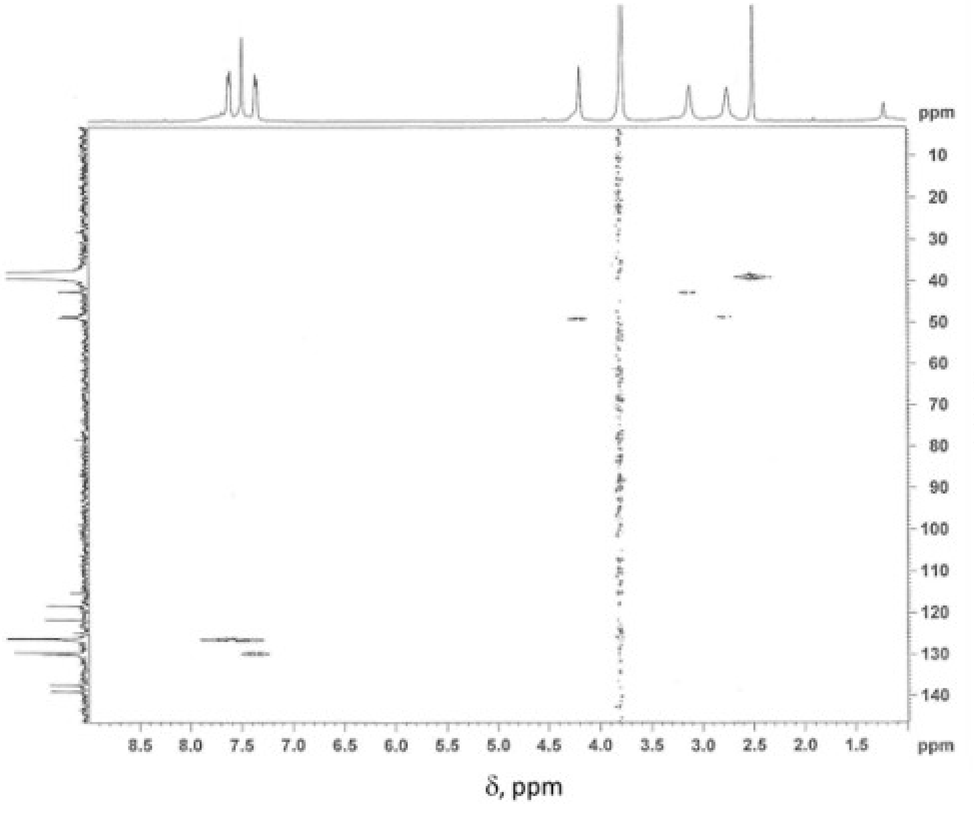

Figure S17. ^{1}H– ^{13}C-HSQC spectrum of L in d_6-DMSO + 1M CF_3SO_3H in D_2O.

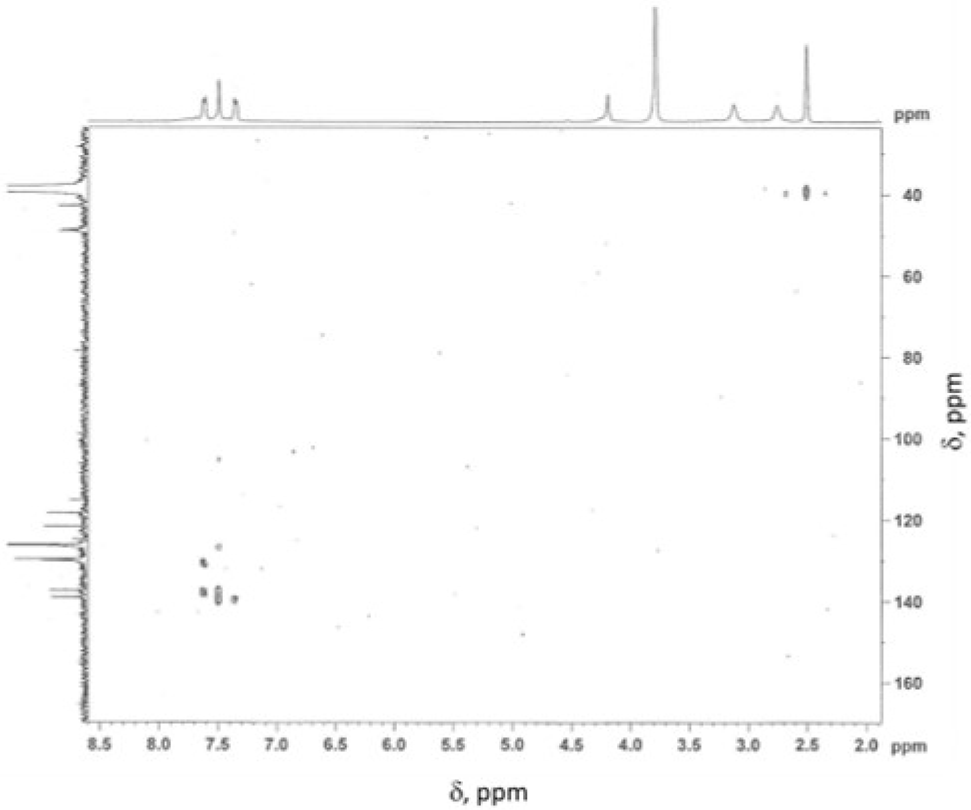

Figure S18. ^1H– ^{13}C-HMBC spectrum of L in d_6-DMSO + 1M CF_3SO_3H in D_2O.

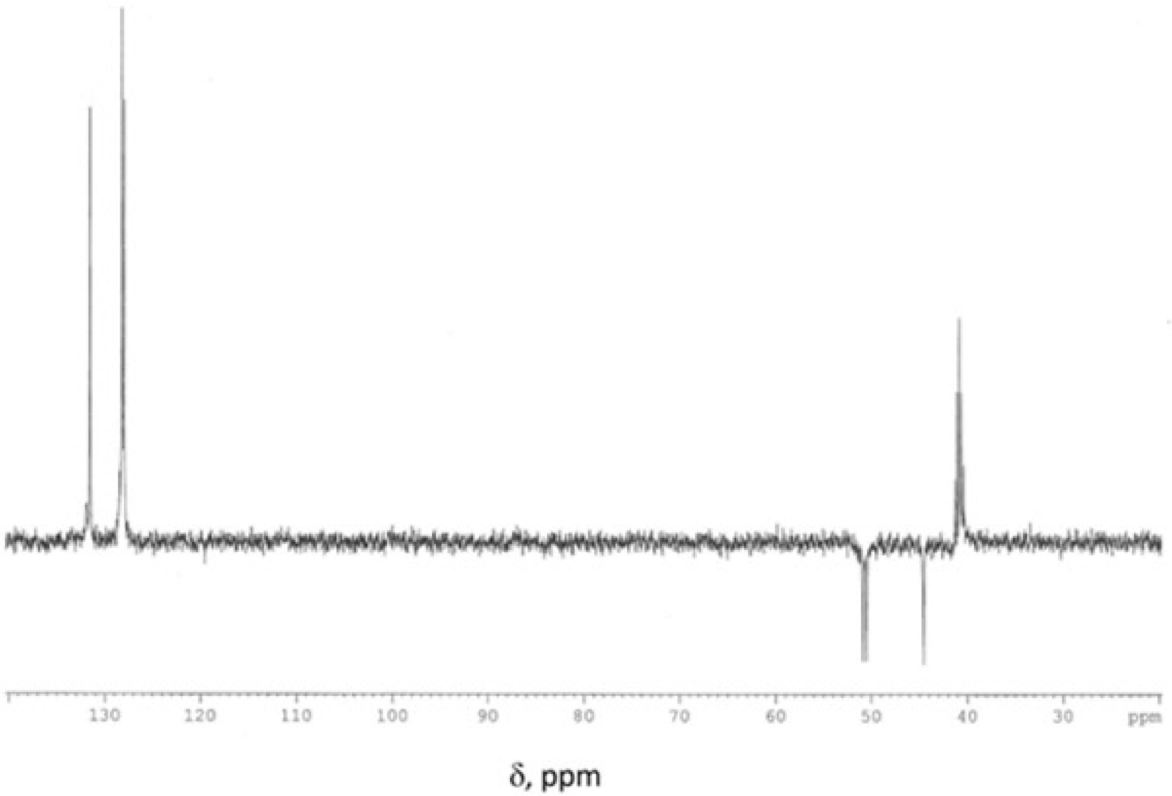

Figure S19. DEPT135 of L in d_6-DMSO + 1M CF_3SO_3H in D_2O.

References

1. Bounos, G.; Ghosh, S.; Lee, A.K.; Plunkett, K.N.; DuBay, K.H.; Bolinger, J.C.; Zhang, R.; Friesner, R.A.; Nuckolls, C.; Reichman, D.R.; et al. Controlling Chain Conformation in Conjugated Polymers Using Defect Inclusion Strategies. *J. Am. Chem. Soc.* **2011**, *133*, 10155–10160; DOI:10.1021/ja2006687.

2. Borch, R.F.; Bernstein, M.D.; Durst, H.D. Cyanohydridoborate anion as a selective reducing agent. *J. Am. Chem. Soc.* **1971**, *93*, 2897–2904; DOI:10.1021/ja00741a013.

3. Gran, G. Determination of the equivalence point in potentiometric titrations. Part II *Analyst* **1952**, 77, 661–671.

4. Gans, P.; Sabatini, A.; Vacca, A. Investigation of equilibria in solution. Determination of equilibrium constants with the HYPERQUAD suite of programs. *Talanta* **1996**, 43, 1739–1753; DOI:10.1016/0039-9140(96)01958-3.

5. Frisch, M.J.; Trucks, J.W.; Schlegel, H.B.; Scuseria, G.E.; Robb, M.A.; Cheeseman, J.R.; Scalmani, G.; Barone, V.; Mennucci, B.; Petersson, G.A.; et al. *Gaussian 09*; Gaussian, Inc.: Wallingford CT, USA, 2009.

6. Gianelli, L.; Amendola, V.; Fabbrizzi, L.; Pallavicini, P.; Mellerio, G.G. Investigation of reduction of Cu(II) complexes in positive-ion mode electrospray mass spectrometry. *Rapid Communications in Mass Spectrometry* **2001**, *15*, 2347–2353; DOI:10.1002/rcm.510.
